# Complications of Cochlear Implant Surgery: A Public Implant Centre Experience

**DOI:** 10.12669/pjms.37.5.3960

**Published:** 2021

**Authors:** Jawwad Ahmed, Ghulam Saqulain, Muhammad Iqbal Javed Khan, Mobeen Kausar

**Affiliations:** 1 Dr. Jawwad Ahmed, FCPS (Otolaryngology) Associate Surgeon, Department of Otolaryngology & Cochlear Implantation, Capital Hospital PGMI, Islamabad, Pakistan; 2 Dr. Ghulam Saqulain, FCPS (Otorhinolaryngology) Head of Department of Otorhinolaryngology & Cochlear Implantation, Capital Hospital PGMI, CDA, Islamabad, Pakistan; 3 Dr. Muhammad Iqbal Javed Khan, FRCS Consultant Otologist & Skull Base Surgeon, Department of Otorhinolaryngology, Bradford Teaching Hospitals NHS Foundation Trust, England; 4 Dr. Mobeen Kausar, MPH Deputy Medical Superintendent, Healthcare Commission Coordinator, DHQ Hospital, Rawalpindi - Pakistan

**Keywords:** Cochlear Implantation, Complications, Hearing Loss, Prevalence

## Abstract

**Objectives:**

To determine the prevalence of complications of cochlear implant surgery in children with congenital profound sensorineural hearing loss.

**Methods:**

This study retrospectively & consecutively reviewed charts of children who underwent cochlear implantation from July 2015 to July 2019 at Cochlear Implant Centre of Otolaryngology Department of Capital Hospital, Islamabad Pakistan. These included cases of both genders aged one to 12 years operated at least one year before the time of data collection. Basic demographic data, complications including major and minor complications and treatment received was noted and statistically analyzed using SPSS-23. Results were presented using descriptive statistics.

**Results:**

Current study included a sample of N=251 having a mean age of 4.05±2.15 years including 154(61.4%) males and 97(38.6%) females revealed a prevalence of complications of 16(6.4%) with 4(1.6%) major and 12(4.8%) minor complications. Wound infection and acute otitis media with frequency of 3(1.2%) each were the commonest complications, followed by , facial nerve twitching, tinnitus and vertigo, infection and extrusion; and device failure in 2(0.8%) each. However, there was no significant association of complications with age group and gender with P=0.344 and P=0.519 respectively.

**Conclusion:**

Present public sector implant program is characterized with a very low prevalence of complications of 16(6.4%) with 4(1.6%) major and 12(4.8%) minor complications. Wound infection and acute otitis media were the commonest complications.

## INTRODUCTION

Severe to Profound Hearing Loss has a prevalence of 6.7% in clinical population and 0.7% in general population as reported for United Kingdom’s National Health Service clinic^1^ and a prevalence of 1.6 per 1000 cases with profound hearing loss alone has been reported from Pakistan.^2^ Cochlear implantation is arguably the best possible option for non-serviceable sensorineural hearing loss (SNHL) with appropriate case selection having paramount importance.^3^ A big population is now receiving cochlear implants with 78% of deaf children receiving cochlear implants in Belgium alone^4^ and according to US Census 2000, there were 12,816 children aged 1 to six years eligible for cochlear implantation.^5^

In cases with severe or profound hearing loss (HL), quite a high prevalence of inner ear malformations (IEMs) of around 15 to 20% has been reported in a study.^6^ Though cochlear implantation in cases with IEMs has favorable surgical and speech results^7^ and is a safe procedure in experienced hands^8^, however difficulties and intraoperative complications do occur.^9^ Complications ranging from pain, minor bleed, infection, breakdown of skin, facial palsy, taste changes, vertigo, cerebrospinal fluid leak, device failure as well as mal-placement, skull base and brain damage to complete deafness and death may occur.^3^ Hence knowledge of associated risks is very important since it is not a lifesaving procedure rather it is responsible for reduction of disability and hence significantly improves quality of life (QoL).^10^

Cochlear implant surgery started in Pakistan in the year 2000, in the private sector alone and the only available small published study gave a complication rate of 11.5% for private sector program.^11^ With a high prevalence of congenital hearing loss in Pakistan^12^, dearth of local literature on the subject and recent development of cochlear implant facility in the public sector in Pakistan was the stimulus to conduct this study on a larger population hailing from all over the country with the objective determine the prevalence of complications of cochlear implant surgery in children with congenital profound sensorineural hearing loss.

This study is important since it will cover the local literature gap being the only study of its type in Pakistan and will be helpful in provision of reliable local data regarding complications of cochlear implantation in patients with congenital HL and planning of effective management strategies.

## METHODS

This study retrospectively conducted chart reviews of children who consecutively underwent cochlear implantation for congenital sensorineural hearing loss, over a period of four years from July 2015 to July 2019. These included cases of both genders aged one to 12 years who were operated at Cochlear Implant Centre of Otolaryngology Department of Capital Hospital, Islamabad Pakistan. All cases were more than one year postoperative at the time of data collection, hence cases operated after 30^th^ June 2019 were not included in the study. Basic demographic data, complications including major and minor complications and treatment received was noted.

Complication of Cochlear Implant surgery was labelled when there was any medical issue arising due to the surgery, which was not expected, resulting in increased morbidity or mandating addition surgical procedure.^13^ Also these were classified as major when there was significant medical problem requiring removal of implant, re-exploration, facial nerve paralysis or paresis or other complication which was serious with continuous dysfunction or discomfort; while minor complications were those requiring no intervention or minor one, resolve by themselves, or easily overcome by little medical or audiologists management.^14^

Study was initiated after obtaining ethical approval of the research from Ethical Review Committee of Capital Hospital PGMI, Islamabad vide Registeration No. 2020-03-003 dated 10^th^ March, 2020. Following data collection, tabulation was done in Microsoft Excel and statistical analyzed performed using Statistical Package for Social Studies (SPSS) Version-23. Statistical analyzed were done using descriptive statistic including frequencies, percentages, mean, and standard deviation. Chi-square test was used for assessment of age and gender association. Data was further examined besides national and international literature and deductions made were finally discussed.

## RESULTS

**Fig.1:**
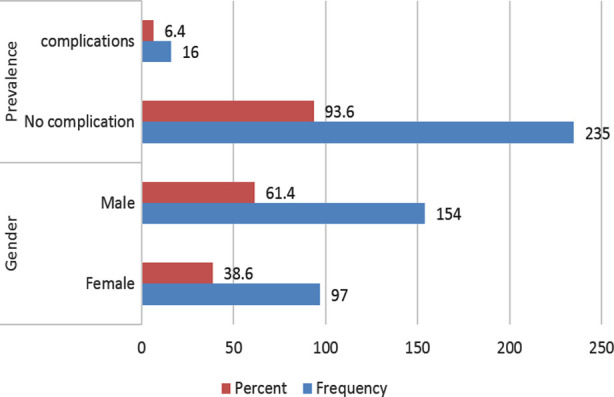
Gender Distribution and Prevalence of Complications (n=251).

N=251 cases of cochlear implantations performed at this public Cochlear implant Centre rom July 2015 to July 2019, were included in the study. With a mean age of 4.05±2.15 years, the study population comprised 154(61.4%) males and 97(38.6%) females. The prevalence of complications was 16(6.4%) (Fig.1).

Minor complications were predominant with frequency of 12(4.8%), followed by major complications 4(1.6%). Wound infection and Acute otitis media predominated with frequency of 3(1.2%) each; followed by facial nerve twitching and Tinitus & vertigo with frequency of 2(0.8%); and Pain/headache & mastoiditis being least common with frequency of 1(0.4%) each (Table-I). Major complications included implant infection & extrusion and Device failure in 2 (0.8%) cases each. There was no significant association of complications with age group and gender with P=0.344 and P=0.519 respectively.

**Table-I:** Complications * Age Group & Gender. Cross Tabulation (n-251).

Complications (N, %)		Age Group			Gender		
	
		>1-3 (81)	>3-5 (144)	>5-18 (25)	>18 Yr (1)	Female (97)	Male (154)
No complication (235, 93.6%)		74	138	22	1	92	143
Major Complications 4(1.6%)	Implant infection & extrusion (2, 0.8)	2	0	0	0	0	2
	Device failure (2, 0.8)	0	2	0	0	0	2
	Facial Nerve Twitching (2, 0.8)	1	1	0	0	0	2
	Wound Infection (3, 1.2)	2	0	1	0	2	1
Minor Complications 12(4.8%)	AOM (3, 1.2)	2	1	0	0	1	2
	Pain/Headache (1, 0.4)	0	0	1	0	0	1
	Mastoiditis (1, 0.4)	0	1	0	0	1	0
	Tinnitus & Vertigo (2, 0.8)	0	1	1	0	1	1
Chi-Square (value,P-value)			26.178, 0.344			7.160, 0.519	

## DISCUSSION

Current study being the largest study from Pakistan involving 251 cases of Government funded cochlear implant surgeries in the public sector revealed a prevalence of complications related to cochlear implant surgery among one to 12 year old children being 16(6.4%) with no death report and with 4(1.6%) major complications and 12(4.8%) minor complications. In contrast an Egyptian study by Sefein IK^15^, revealed a high complication rate of 18.75% with 8.03% minor and 10.71% major complications with no death. Another study by Farinetti A et al. also reported a high complication rate of 19.9% with minor complications in 14.9% and major in 5% cases. They also noted significant higher complication rate in adults.^16^ Achiques MT has reported a prevalence of 11.38%.^17^ While, Awad AH et al^18^ reported complication rate of 10.43% with 6.75% minor and 3.68% major complications. Dodson KM et al reported a complication rate of 9.3%, with 59% related to device failure and 3 intracranial complications including two with minor dura leak which was repaired with fascia and one with acute hematoma treated by evacuation.^19^

In a previous study conducted in Pakistan, in the private sector comprised of only 52 cases and revealed much higher prevalence of complications of 11.5% with 3.8% failure rate of the device.^11^ Current study has the advantage of being conducted for a population in which implant and surgery was government funded resulting in a sample hailing for all provinces and areas of Paksitan, while the previous study was in the private sector in which funding was a substantial issue.^11^ The difference in the complication rate of the two studies might be due to better audiological, surgical and follow up of cases in current study. In a study by Saunders & Barrs^20^, to make recommendations for implantation in developing countries, while studying challenges in developing nations, authors reported that most respondents (83%) agreed that audiologist was most important requirement and limitation of audiological services was the most important postop concern compared to postoperative care and infection. It must also be noted that over the years, Pakistan has developed better audiological services. In quite similarity to our study, Binnetoglu A et al. in a large study with 2597 cases and mean age of 6.48 years reported a very low complication rate of 3.7% with 3% minor and 0.7% major complications.^21^ However in study bin Binnetoglu A et al. vertigo was the commonest minor complication while implant extrusion was the major complication and authors supported the closure of the cochleostomy or round window with muscle to avoid complications.^21^ In present study also among major complications implant infection & extrusion and device failure were noted among the major complications. To avoid electrode extrusion and or migration, Kubo T et al. have also advocated tight packing of cochleostomy, split bridge technique and canal wall reconstruction.^22^ It has also been highlighted that vertigo with non-improvement in hearing should arouse suspicion of mal-placement of electrodes.^23^ Also in a study by Yi J et al.^24^, out of 1065 implantations 28 (2.63%) developed complications with 7 (0.66%) major and 21 (1.97%) minor and only two re-implantations were required with no case of severe infection, necrosis of flap or extrusion of device. The Major complications included magnet and electrode displacement, traumatic failure of implant and minor CSF leak.^24^ High frequency of complications of 13.25 was reported by Júnior LRPL et al. including 8% minor and 5.2% major complications.^25^ Compared to these, the current study had a low prevalence of 4(1.6%) major and 12(4.8%) minor complications and with regards individual complications of surgery, Wound infection and Acute Otitis Media were the commonest complications noted in 3(1.2%) each. Facial nerve twitching/ stimulation occurred in 2(0.8%). Implant infection & extrusion and device failure occurred in 2(0.8%) each. Sefein IK reported 12 cases with major complications including misplaced electrodes in two, cerebrospinal fluid leak in four, labyrinthitis ossificans, magnet displacement, central perforation, seroma and hematoma in, wound infection, and persistent pain/discomfort in one case each.^15^ Study by Farinetti A et al.^16^ and Awad AH et al.^18^ also revealed that in children infections like AOM were the commonest complication, while common major complication was device failure. While, Halawani R et al. for a sample of adults and children reported a complication rate of 10.2% with 9.5% being minor with hematoma/ seroma being commonest complication.^26^ Other studies have also reported hematoma, infection^22^, and device failure^17^ as the commonest complications.^25^ Infection can even progress to meningitis, however most respond to medications, however skin breakdown may warrant surgical intervention.^22^ A Saudi study by Al Shaikh AM et al.^27^, revealed a prevalence of 11.4% in children including 7.7% major and 4.8% minor complications including spontaneous device failure 2.8%, traumatic device failure 2.1%, Wound infection and dehiscence 1.4% each, hematoma 0.7%, post-operative vertigo, facial twitching, and otitis media in 0 .7% each.

Some other complications may also rarely occur. Facial Nerve stimulation occurred in just 0.8% of our cases and was not related to IEM’s, According to Kubo T et al this commonly occurs in ears with anomalies and can be dealt with reprogramming some electrodes, however decrement in speech recognition may occur.^22^ Rarely, auditory nerve may give poor response as a result patient becomes aware of only environmental sounds and avoids implant use.^22^ Also rarely complications like periorbital ecchymosis can occur, hence avoidance of undue extensive periosteal elevation and dissection of other subcutaneous tissues.^28^

Depending on specific individual anatomical considerations of patient preoperative planning and adoption of soft surgical techniques result in reduction of surgical complications.^24^ Preoperative protocol followed, safe surgical techniques and post-operative care are responsible for reduction in complications.^26^ Hence continued surgeon education and training is emphasized.^25^

## CONCLUSIONS

Present public sector implant program is characterized with a very low prevalence of complications of 16(6.4%) with 4(1.6%) major and 12(4.8%) minor complications. Wound infection and acute otitis media were the commonest complications.

### Authors Contribution:

**JA:** Did the data collection, analysis and interpretation of results.

**GS:** Was responsible for conception of work, writing of manuscript and is responsible for integrity of research

**IJK:** Did the critical revision of the manuscript

**MK:** Did the literature review

## References

[1] Turton L, Smith P (2013). Prevalence &characteristics of severe and profound hearing loss in adults in a UK National Health Service clinic. Int J Audiol.

[2] Ali G (2010). Genetic deafness in Pakistani Population. J Pak Med Assoc.

[3] Krogmann RJ, Al Khalili Y (2020). Cochlear Implants. In:StatPearls [Internet].

[4] De Raeve L (2016). Cochlear implants in Belgium:Prevalence in paediatric and adult cochlear implantation. Ann Otorhinolaryngol Head Neck Dis.

[5] Bradham T, Jones J (2008). Cochlear implant candidacy in the United States:Prevalence in children 12 months to 6 years of age. Int. J. Pediatr. Otorhinolaryngol.

[6] Quirk B, Youssef A, Ganau M, D'Arco F (2019). Radiological diagnosis of the inner ear malformations in children with sensorineural hearing loss. Brit J Radiol.

[7] Farhood Z, Nguyen SA, Miller SC, Holcomb MA, Meyer TA, Rizk HG (2017). Cochlear Implantation in Inner Ear Malformations:Systematic Review of Speech Perception Outcomes and Intraoperative Findings. Otolaryngol. Head Neck Surg.

[8] Terry B, Kelt RE, Jeyakumar A (2015). Delayed complications after cochlear implantation. JAMA Otolaryngol Head Neck Surg.

[9] Woolley AL, Jenison V, Stroer BS, Lusk RP, Bahadori RS, Wippold FJ (1998). Cochlear implantation in children with inner ear malformations. Ann Otol Rhinol Laryngol.

[10] McRackan TR, Bauschard M, Hatch JL, Tobin EF, Droghini HR, Nguyen SA (2018). Meta-Analysis of Quality-of-Life Improvement after Cochlear Implantation and Associations With Speech Recognition Abilities. Laryngoscope.

[11] Khan MIJ, Mukhtar N, Saeed SR, Ramsden R (2007). The Pakistan (Lahore) cochlear implant programme:Issues relating to implantation in a developing country.

[12] Ahmed S, Sheraz S, Malik SA, Ahmed NR, Malik SA, Farooq S (2018). Frequency of Congenital Hearing Loss in Neonates. J Ayub Med Coll Abbottabad.

[13] Hansen S, Anthonsen K, Stangerup SE, Jensen JH, Thomsen J, Caye-Thomasen P (2010). Unexpected findings and surgical complications in 505 consecutive cochlear implantations:a proposal for reporting consensus. Acta Otolaryngol.

[14] Sanna M, Free R, Merkus P, Falcioni M, Caruso A, Donato GD, Complications and Revision Surgery in Cochlear Implantation Surgery for Cochlear and Other Auditory Implants. New York:Thieme.

[15] Sefein IK (2018). Surgical complications and morbidity in cochlear implantation. Egypt J Otolaryngol.

[16] Farinetti A, Mancini J, Ben Gharbia D, Roman S, Nicollas R, Triglia JM (2014). Les complications de l'implant cochléaire chez 403 patients:etude comparative adulte-enfant et revue de la littérature. Annales françaises d'Oto-rhino-laryngologie et de Pathologie Cervico-faciale.

[17] Achiques MT, Morant A, Munoz N, Marco J, Llopez I, Latorre E (2010). Cochlear implant complications and failures. Acta Otorrinolaringol Esp.

[18] Awad AH, Rashad UM, Gamal N, Youssif MA (2018). (2018) Surgical complications of cochlear implantation in a tertiary university hospital Cochlear. Implants Int.

[19] Dodson KM, Maiberger PG, Sismanis A (2007). Intracranial Complications of Cochlear Implantation. Otol. Neurotol.

[20] Saunders J, Barrs D (2011). Cochlear Implantation in Developing Countries as Humanitarian Service:Physician Attitudes and Recommendations for Best Practice. Otolaryngol. Head Neck Surg.

[21] Binnetoglu A, Demir B, Batman C (2020). Surgical complications of cochlear implantation:a 25-year retrospective analysis of cases in a tertiary academic center. Eur Arch Otorhinolaryngol.

[22] Kubo T, Matsuura S, Iwaki T (2005). Complications of cochlear implant surgery. Oper Tech Otolayngol Head Neck Surg.

[23] Mecca MA, Wagle W, Lupinetti A, Parnes S (2003). Complication of Cochlear Implantation Surgery. Am J Neuroradiol.

[24] Jiang Y, Gu P, Li B, Gao X, Sun B, Song Y (2017). Analysis and Management of Complications in a Cohort of 1,065 Minimally Invasive Cochlear Implantations. Otol Neurotol.

[25] Junior LRPL, Junior FDAR, Calhau CMDF, Calhau ACDF, Palhano CTDP (2010). Postoperative Complications in implanted patients in the CochlearImplant Program of Rio Grande do Norte –Brazil. Braz J Otorhinolaryngol.

[26] Halawani R, Aldhafeeri A, Alajlan S, Alzhrani F (2019). Complications of post-cochlear implantation in 1027 adults and children. Ann Saudi Med.

[27] Al Shaikh AM, Aljedaani Y, Al Essa MA, Hassen HE (2016). Complications of cochlear implantations. Saudi J Otorhinolaryngol Head Neck Surg.

[28] Gyanwali B, Tang J, Li H, Tang A (2016). Periorbital Ecchymosis:An Uncommon Complication of Cochlear Implantation. Anat Physiol.

